# Clinical and Radiologic Features Correlated with Eye-Sparing Surgery vs. Orbital Exenteration for Lacrimal Gland Adenoid Cystic Carcinoma †

**DOI:** 10.3390/cancers18182911

**Published:** 2026-09-09

**Authors:** Bita Esmaeli, Yunyi Wang, Tracy Lu, Jing Ning, Amy C. Moreno, Steven J. Frank, Luana G. Sousa, Renata Ferrarotto, J. Matthew Debnam

**Affiliations:** 1 Department of Ophthalmology, Houston Methodist Hospital Academic Institute, Houston, TX 77030, USA; 2 Oncologic Ophthalmic Plastic & Orbital Surgery, Mann Eye Institute, Houston, TX 77004, USA; 3 Department of Clinical Sciences, University of Houston Tilman J. Fertitta Family College of Medicine, Houston, TX 77001, USA; 4 Department of Biostatistics, The University of Texas MD Anderson Cancer Center, Houston, TX 77030, USA; 5 Orbital Oncology and Ophthalmic Plastic Surgery, Department of Plastic Surgery, The University of Texas MD Anderson Cancer Center, Houston, TX 77030, USA; 6 Department of Radiation Oncology, The University of Texas MD Anderson Cancer Center, Houston, TX 77030, USA; 7 Department of Head and Neck & Thoracic Medical Oncology, The University of Texas MD Anderson Cancer Center, Houston, TX 77030, USA; 8 Department of Neuroradiology, The University of Texas MD Anderson Cancer Center, Houston, TX 77030, USA

**Keywords:** lacrimal gland adenoid cystic carcinoma, orbital exenteration, eye sparing surgery, globe sparing surgery, crossing the vertical midline, T category

## Abstract

**Background**: To determine if certain clinical and radiologic features of lacrimal gland adenoid cystic carcinoma (LGACC) on pre-operative MRI were associated with the treatment decision to perform orbital exenteration versus eye-sparing surgery. **Methods**: All consecutive patients with LGACC treated by orbital exenteration (Group 1) versus eye-sparing surgery (Group 2) from June 2007 to March 2024 were identified through a search of the primary author’s patient database. Data analyzed included patient demographics, T category at presentation (using AJCC 8th edition criteria), and magnetic resonance imaging (MRI) features, including T1 post-contrast enhancement patterns and T2 signal characteristics. The MRIs were reviewed by a CAQ-certified neuroradiologist. The location of the LGACC—including the distance of the posterior tumor margin from the superior orbital fissure (SOF), bone remodeling and destruction, involvement of the extra-ocular muscles (EOMs), temporal fossa, pterygopalatine fossa, sinonasal cavity, intracranial compartment, and the presence of perineural tumor spread—were analyzed. **Results**: The study included a total of 39 patients. A total of 16 patients with a median age of 52.6 years were in Group 1, and 23 patients with a median age of 46.1 years were in Group 2. A greater T-category and larger size of the tumor were significantly more common in Group 1. Smooth tumor margins were significantly more common in Group 2, while irregular margins were significantly more common in Group 1. The distance from the posterior tumor margin to the SOF was significantly less in Group 1. Crossing the vertical midline of the orbit, bone destruction, and the invasion of the EOMs were significantly more common in Group 1. Extension to involve the cavernous sinus, pterygopalatine foramen, and sinonasal cavity, as well as perineural spread, were only present in Group 1. No significant difference was noted between T1 post-contrast patterns and T2 characteristics. **Conclusions**: MRI features of LGACC more commonly associated with orbital exenteration as opposed to eye-sparing surgery included larger size and irregular margins, as well as the posterior tumor margin being closer to the SOF, crossing the vertical midline of the orbit, bone destruction, and the involvement of the EOMS, cavernous sinus, pterygopalatine foramen, and sinonasal cavity. These MRI features may aid oculoplastic surgeons in determining which patients may be candidates for successful eye-sparing surgery for the treatment of LGACC.

## Simple Summary

The management of lacrimal gland carcinomas has evolved in the last two decades with a shift towards eye-sparing surgery followed by radiation therapy for most patients. However, there is lack of accepted criteria for the selection of patients for eye-sparing surgery vs. orbital exenteration. Our study highlights the clinical and radiologic characteristics that correlate with the choice of surgery. Larger tumor size, greater T category, irregular margins, posterior tumor extension, the involvement of the extraocular muscles, and the extension of tumor across the vertical midline of the orbit correlated with the need for orbital exenteration. These MRI features may aid oculoplastic surgeons in determining which patients may be candidates for successful eye-sparing surgery vs. those who may need to have an orbital exenteration or might benefit from neoadjuvant attempts at tumor shrinkage prior to surgery.

## 1. Introduction

Primary tumors of the lacrimal gland are rare, occurring in less than 1 per 1,000,000 people per year [1]. The most common malignant epithelial tumor of the lacrimal gland is adenoid cystic carcinoma, accounting for approximately 75% of the lacrimal gland carcinomas in most series [2].

Although historically, orbital exenteration was offered as the primary “standard” surgical modality for this rare cancer [3,4,5], more recent reports have increasingly focused on and advocated for newer treatment approaches consisting of eye-sparing surgery, defined as the gross total resection of the lacrimal gland mass with only microscopic disease remaining, followed by radiotherapy and/or concurrent chemotherapy and radiation therapy [6,7,8,9,10].

The rare nature of LGACC makes prospective trials and head-to-head comparisons of various treatments impossible. Most reports contain relatively few patients with anywhere from 11 to 52 patients treated at single centers with various modalities [2,3,4,5,6,7,8,9,10].

In a recent single-center report of 52 patients with lacrimal gland adenoid cystic carcinoma (LGACC), which is one of the largest series reported to date, we reported that about 50% of patients had eye-sparing surgery, and the rest had orbital exenteration. Furthermore, in the same report, we found that, since the availability of eye-sparing surgery approaches at our center in 2007, about 75% of patients had eye-sparing surgery and only 25% had exenteration [10]. According to several publications from multiple centers, the local control rates seem to be comparable in LGACC patients who have eye-sparing surgery followed by radiation compared with those who have orbital exenteration followed by radiation [6,7,8,9,10]. The multi-modality eye-sparing approach outlined in some of the recent reports seem to also suggest favorable survival with 5- and 10-year DSS rates as high as 75% and 60%, respectively, which are better compared to historical reports [10]. Furthermore, several reports have suggested comparable survival rates between patients who have eye-sparing surgery followed by radiation therapy compared with cohorts that have various forms of neoadjuvant and adjuvant chemotherapy combined with orbital exenteration and adjuvant radiation therapy [8,11].

In the current report, we aim to test the hypothesis that certain clinical and radiological features such as the size of the lacrimal gland mass, posterior extension of the carcinoma, AJCC T category at presentation, and involvement of the extraocular muscles may be correlated with the ability to offer eye-sparing surgery vs. orbital exenteration as the definitive surgery in patients with LGACC.

## 2. Patients and Methods

All consecutive patients who had a diagnosis of lacrimal gland adenoid cystic carcinoma and were treated by the primary author (BE) during the period from June 2007 to March 2024 were included. The medical records, including radiologic records, of all eligible patients were retrospectively reviewed. The eligibility criteria for inclusion in the study was a diagnosis of primary lacrimal gland adenoid cystic carcinoma as confirmed based on surgical pathology. Only patients for whom reliable radiologic images before surgery were available for analysis were included. The clinical parameters analyzed included age, gender, date of initial diagnosis, T category at presentation using the 8th edition of AJCC, and histopathologic diagnosis.

The radiologic data analyzed included radiologic findings on the initial magnetic resonance imaging (MRI) and/or computed tomography (CT). The imaging studies were reviewed by a CAQ-certified neuroradiologist with over 25 years of experience. Tumor size and distance from the posterior margin of the lacrimal gland carcinoma to superior orbital fissure (SOF) were measured in each case. Other features that were specifically evaluated included whether the extraocular muscles were infiltrated by tumor; extension beyond the bony walls of the orbit; crossing the vertical midline of the orbit; and the involvement of extra-orbital structures such as pterygopalatine fossa, temporal fossa and paranasal sinuses. The enhancement pattern (homogeneous; heterogeneous solid components > 50% enhancement; heterogeneous solid components < 50% enhancement; or peripheral enhancement) and T2 signal characteristics (hypointense, isointense, and/or hyperintense to muscle) were recorded.

Group 1 patients were defined as patients who had orbital exenteration as the definitive surgery for LGACC. Group 2 patients were defined as those who had eye-sparing surgery as the definitive surgery for LGACC. Any neoadjuvant or adjuvant treatments such as chemotherapy and radiation therapy were recorded in each case. All measured variables were compared between Group 1 and Group 2.

This research was done in accordance with the Health Insurance Portability and Accountability Act. Institutional Review Board approval was obtained from The University of Texas MD Anderson Cancer Center (protocol number: PA 2024-1244). This research adhered to the tenets of the Declaration of Helsinki. The requirement for informed consent was waived since this is not an interventional study and the retrospective data review is of minimal risk to patients.

### Statistical Methods

Descriptive statistics were used to describe the patients’ characteristics and clinical outcomes. Continuous variables were summarized using mean, median, interquartile range (IQR), and range, while categorical variables were described by frequencies and percentages.

The MRI features were summarized using descriptive statistics. To compare these variables between the two groups, two-sample *t*-tests were used to compare continuous variables following normal distribution (age and size), the Wilcoxon rank sum test was used to compare continuous variables not following normal distribution (e.g., distance between posterior tumor margin and SOF), and Fisher’s exact tests were used for categorical variables. *p*-values of less than 0.05 were considered statistically significant. Statistical analyses were conducted in R (version 4.3.1) [12].

## 3. Results

The study included a total of 39 patients. A total of 16 patients with a median age of 52.6 years (range: 32–70) were in Group 1, and 23 patients with a median age of 46.1 years (range: 13–73) were in Group 2.

The clinical and radiologic features for Group 1 vs. Group 2 are summarized in Table 1 with statistically significant differences highlighted in bold.

**Figure 1:**
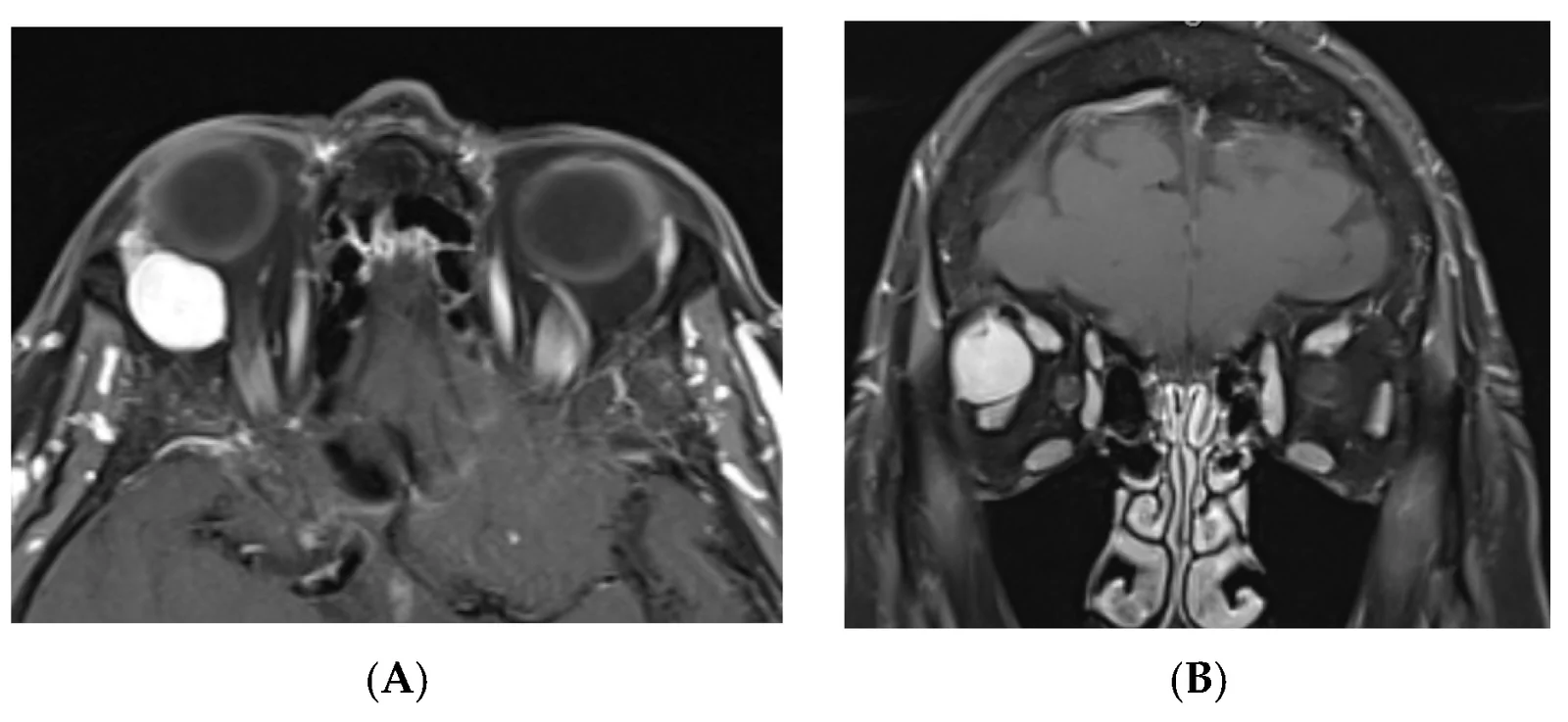
A 71-year-old woman presented with complaint of fullness in the upper eyelid without pain. (**A**,**B**) MRI images, axial (**A**) and coronal (**B**), T1 post-contrast images show a round, well-circumscribed lesion of the lacrimal gland. On gross total resection, the lesion turned out to be adenoid cystic carcinoma. The patient had post-op adjuvant radiation therapy.

**Figure 2:**
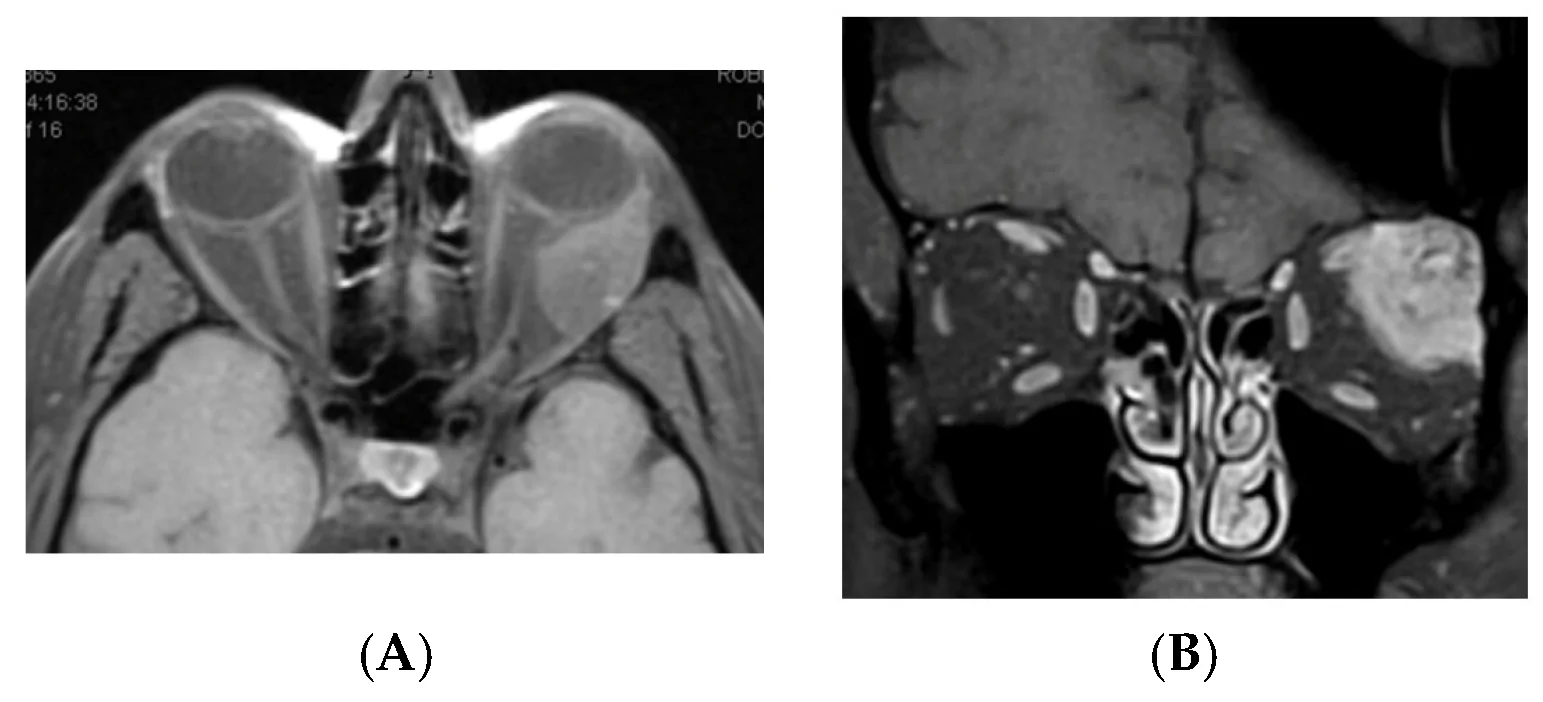
**A** 60-year-old man presented with 6 months history of progressive painless proptosis of the left eye. (**A**,**B**) MRI axial (**A**) and coronal (**B**) T1 weighted post-contrast fat-suppressed images show a left lacrimal gland mass. An incisional biopsy confirmed lacrimal gland ACC. The patient subsequently had eye-sparing gross total resection of the left lacrimal gland mass followed by adjuvant radiation therapy.

**Figure 3:**
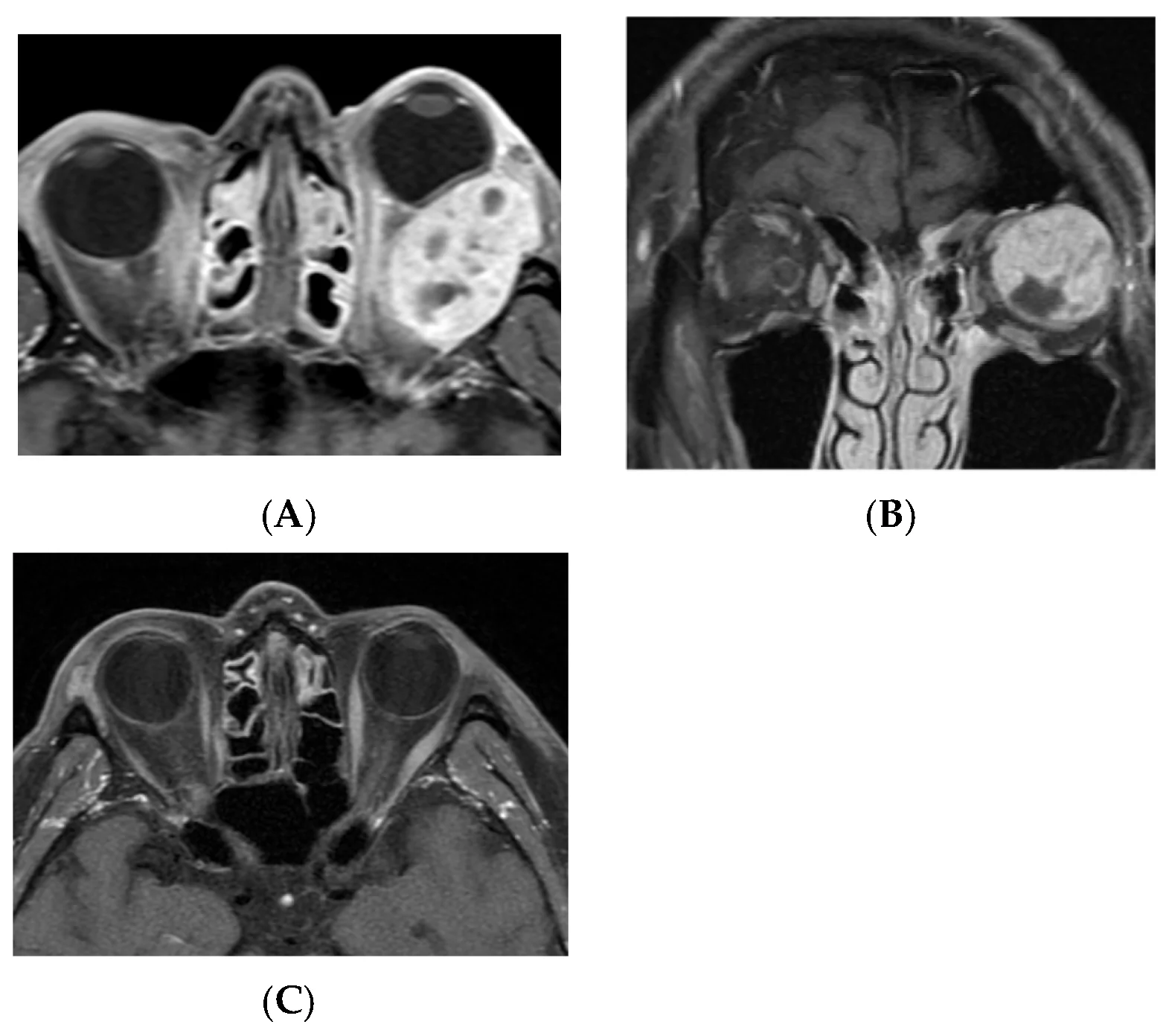
A 55-year-old man presented with a history of transient diplopia and progressive proptosis for the past 3 years with increasing “pressure sensation” in the last three months. On examination, he had 10 mm of proptosis, restriction in left gaze and some visual loss due to choroidal striae. (**A**,**B**) MRI axial (**A**) and coronal (**B**) T1 weighted post-contrast fat-suppressed images show a 3.6 × 2.9 cm mass arising from the lacrimal gland and very closely associated with the belly of the left lateral rectus muscle. There are compression and anteromedial displacement of the optic nerve and proptosis of the left eye. The patient had eye-sparing surgery: gross total resection of the lacrimal gland mass, followed by post-operative adjuvant radiation therapy. (**C**) MRI, 25 months after surgical resection of the lacrimal gland ACC and adjuvant radiation therapy, shows no evidence of local recurrence.

**Figure 4:**
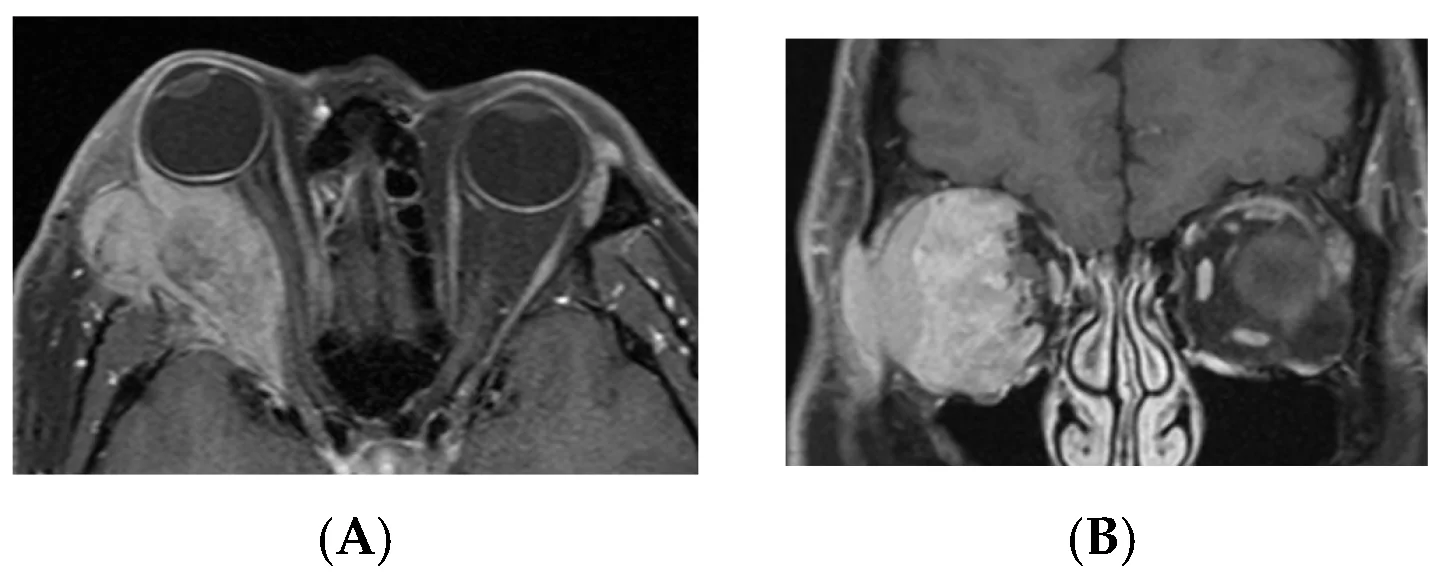
A 41-year-old man presented with about 6–8 months history of progressive proptosis and pain in the right orbital area. An incisional biopsy prior to referral confirmed the diagnosis of adenoid cystic carcinoma. (**A**,**B**) MRI findings, axial (**A**) and coronal (**B**) T1 weighted post-contrast fat-suppressed images show a hypointense homogeneously enhancing mass centered at the right lateral orbit measuring approximately 4.2 × 5.9 × 4.3 cm. Laterally, this mass invades the lateral orbital wall with invasion of the anterior aspect of the temporalis muscle. There is also involvement of the greater sphenoid wing, frontal process of the sphenoid, orbital floor, and orbital roof. Posteriorly, the mass extends to the orbital apex with involvement of the infraorbital foramen. There is infiltration of all extraocular muscles except for the medial rectus muscle. This patient had an orbital exenteration including resection of the sphenoid bone and extra-orbital component of the lesion followed by a free flap reconstruction and post-operative adjuvant radiation therapy.

**Figure 5:**
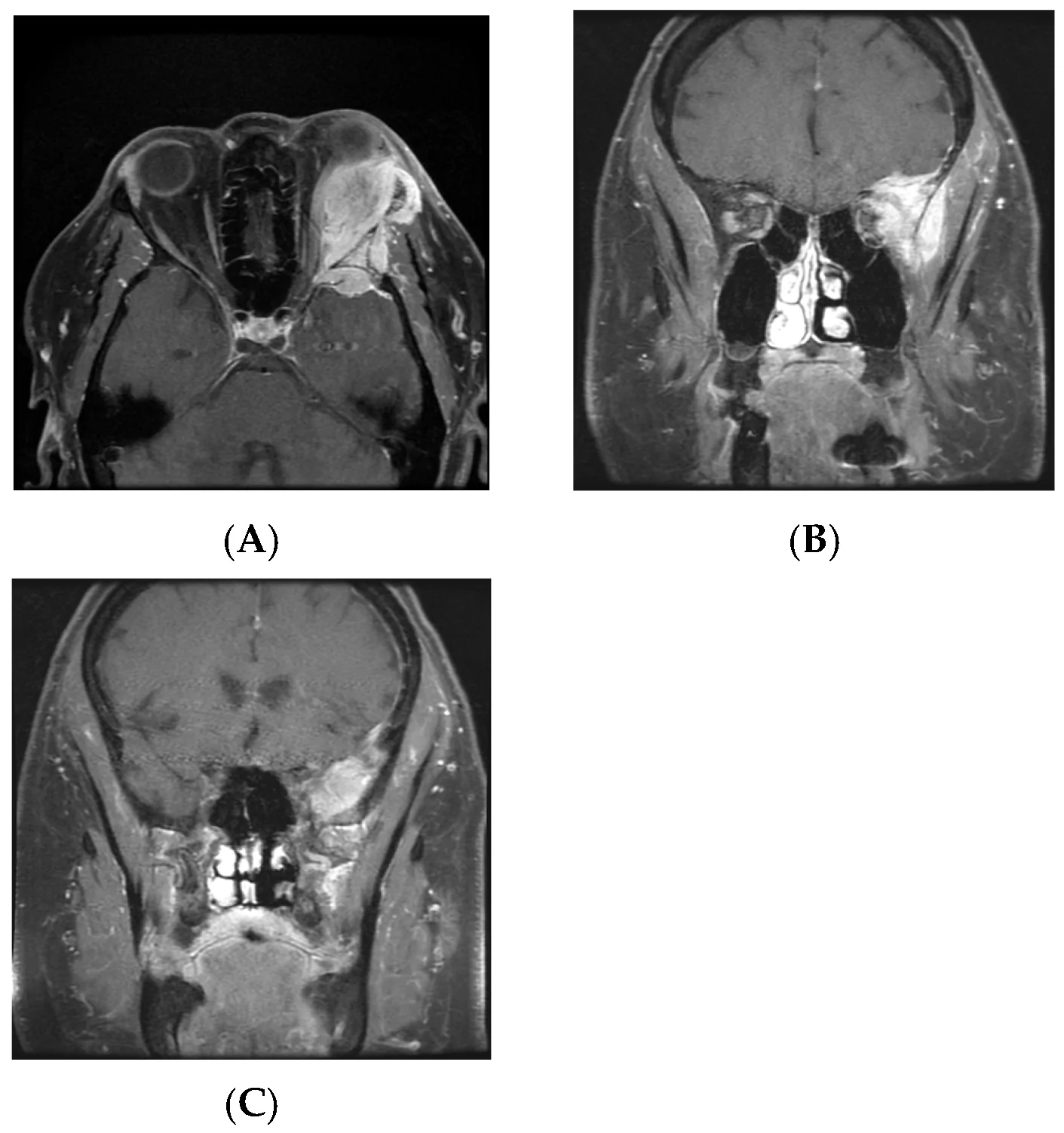
A 64-year-old man presented with progressive proptosis and complained of pain. An incisional biopsy prior to referral had confirmed the diagnosis of LGACC. (**A**–**C**) MRI findings on axial (**A**) and two coronal images (**B**,**C**) show a locally advanced adenoid cystic carcinoma of the left lacrimal gland with extension to the dura of the temporal lobe, sphenoid bone, and temporal fossa. Neoadjuvant intraarterial chemotherapy was given without any measurable response. The patient had an orbital exenteration through a combined frontal and cranial approach, with a modified orbital zygomatic craniotomy for approach to middle cranial fossa and infratemporal fossa with resection of middle cranial fossa tumor extension. He had a free flap reconstruction for the defect and adjuvant post-operative radiation therapy.

Significant differences were found between Group 1 and Group 2. A greater T category and larger size of the tumor were significantly more common in Group 1 (average tumor size in Group 1: 3.8 cm vs. Group 2: 2.9 cm). Smooth tumor margins were significantly more common in Group 2, while irregular margins were significantly more common in Group 1. The distance from the posterior tumor margin to the SOF was significantly less in Group 1 (median distance in Group 1: 7.5 mm; (range: 0–23) vs. 15 mm (range: 0–41) for Group 2. Crossing the vertical midline of the orbit, the erosion of the bony walls of the orbit, the involvement of the intraconal space, and the invasion of the EOMs were significantly more common in Group 1 compared to Group 2. Tumor extension to involve the cavernous sinus, pterygopalatine foramen, and sinonasal cavity, as well as perineural spread, were only present in Group 1 patients and not seen at all in Group 2 patients. Imaging findings as examples of Group 1 and Group 2 patients are shown in Figure 1, Figure 2, Figure 3, Figure 4 and Figure 5, respectively. No significant differences were noted between T1 post-contrast enhancement patterns and T2 signal characteristics between the two groups.

## 4. Discussion

The management of lacrimal gland carcinomas has evolved significantly in the last two decades with a shift towards the consideration of eye-sparing surgery followed by radiation therapy for most patients [2,6,7,8,9,10,13,14,15,16,17,18,19,20,21,22,23,24,25,26,27,28,29,30,31]. This shift in perspective has been supported by several publications from various centers throughout the world [2,6,7,8,9,10]. However, even in the era of the acceptance of eye-sparing approaches, not every patient with lacrimal gland carcinoma would be appropriate for eye-sparing surgery, and there is a lack of accepted criteria for the selection of patients for eye-sparing surgery vs. orbital exenteration. Our report highlights the clinical and radiologic characteristics that may help the treating orbital surgeon in selecting patients for eye-sparing surgery vs. orbital exenteration. Our findings may further help in the selection of patients who may be good candidates for the consideration of neoadjuvant treatments, such as chemotherapy prior to surgery, with the idea to shrink the lesion and make the possibility of eye-sparing surgery more likely.

The current report is based on retrospective data in a cohort of 39 patients with adenoid cystic carcinoma of the lacrimal gland treated since the year 2007, when eye-sparing surgery followed by adjuvant radiation therapy first became available at our center. Our findings confirm the hypothesis that many clinical and radiologic features correlate with the decision to do eye-sparing surgery vs. orbital exenteration. To our knowledge, this type of correlation has not been previously reported in the literature. Some of the significant findings may be viewed as intuitive, such as the size of the lesion and the T category at presentation, the latter being mostly driven by tumor size and the extent of lesion beyond the orbital bony walls. Other specific features such as the tumor crossing the vertical midline of the orbit, the infiltration of the extraocular muscles, the distance from the posterior margin of the lacrimal gland mass to superior orbital fissure, and the smoothness of the margins of lacrimal gland mass have not been previously studied or reported as important determinants of the type of surgery offered for patients with lacrimal gland ACC. Our findings suggest that these radiologic features should be routinely commented on in radiology reports and carefully considered by the orbital surgeon when deciding on definitive surgery in a patient with a newly diagnosed LGACC.

Extension into the pterygopalatine fossa, cavernous sinus, and paranasal sinuses were seen in a few cases and only in patients who had orbital exenteration (Group 1). It is important to note that these findings were not iatrogenic in nature and highlight the aggressive biologic nature of LGACC if it goes untreated or if attempts at neoadjuvant treatments fail. Some authorities have warned against the removal of bony orbital walls during surgery for LGACC for fear of increased risks of skull base extension by removing the bony barriers. However, the few cases in our series who presented with spontaneous erosion and extension into the skull base or paranasal sinuses remind us that, in such cases, the total resection of the full extent of the mass would likely entail the removal of the bony walls that are infiltrated by tumor. If such patients with skull base and paranasal sinus extension of LGACC also present with systemic metastatic disease at the time of the presentation of the lacrimal gland mass, all treatments would be palliative, and even radical surgery would be done with palliative intent and may be appropriate to prolong life or improve quality of life. Although tumor extension involving the cavernous sinus, pterygopalatine fossa, and sinonasal cavity, as well as perineural spread, were observed only among patients in Group 1, the corresponding comparisons with Group 2 were not statistically significant, likely due to the small sample size. Therefore, these findings should be considered descriptive in nature rather than confirmatory due to the sample size of the study.

It is also important to note that several key characteristics were significantly interrelated. T stage was associated with tumor size (*p* = 0.013), distance from the posterior tumor margin to the SOF (*p* = 0.003), bone erosion (*p* < 0.001), and temporal fossa involvement through the lateral wall (*p* < 0.001). Tumor size was also associated with margins and multiple measures of tumor extension (all *p* ≤ 0.026), while distance from the SOF was associated with all other evaluated features (all *p* ≤ 0.027). These results indicate substantial associations among the evaluated variables; therefore, their individual associations with the choice of surgery from the univariable analyses should not be interpreted as independent effects.

Our findings also have implications for the selection of patients for neoadjuvant treatments, such as intravenous or intra-arterial chemotherapy or targeted therapy, prior to definitive surgery in patients with LGACC [5,11,18]. Such treatments would seem appropriate only in patients with the clinical and radiologic features found in our report to be associated with orbital exenteration (Group 1). Offering neoadjuvant chemotherapy to all patients with LGACC without any consideration of high-risk features correlated with exenteration would seem inappropriate, as it would delay definitive surgery and would unnecessarily expose patients with low-risk features (Group 2) to the side effects of chemotherapy and the potential for further progression of the lesion if chemotherapy is not successful.

The limitation of the current report is its retrospective nature and the relatively small number of patients. LGACC is a very rare cancer, and prospective head-to-head comparison between treatment modalities is not feasible. As the choice of surgery was at the judgement of the senior surgeon, there is inherent bias in this selection. Nevertheless, this cohort of 39 patients from a recent period when both eye-sparing surgery and orbital exenteration have been offered provides an initial attempt at suggested clinical and radiologic features to consider for the surgical management of LGACC and for the selection of patients for neoadjuvant treatments, and to our knowledge, there are no other guidelines to help the treating surgeon make these choices when deciding on eye-sparing surgery vs. orbital exenteration for patients with LGACC.

## 5. Conclusions

Several radiologic features were found to correlate with the choice of orbital exenteration vs. eye-sparing surgery for LGACC. These include the size of the lesion and the T category at presentation, the tumor crossing the vertical midline of the orbit, the infiltration of the extraocular muscles, the distance from the posterior margin of the lacrimal gland mass to superior orbital fissure, and the smoothness of the margins of lacrimal gland mass. These radiologic features should be routinely commented on in radiology reports and carefully considered by the orbital surgeon when deciding on definitive surgery and also for the selection of patients with a newly diagnosed LGACC for neoadjuvant chemotherapy.

## Figures and Tables

**Table 1:** Clinical and radiologic characteristics of Group 1 (orbital exenteration) vs. Group 2 (eye-sparing surgery).

Characteristic	Group 1: Orbital Exenteration (N = 16)	Group 2: Eye-Sparing Surgery (N = 23)	Total (N = 39)	*p*-Value
**Age (years)**				0.459
Mean	51.2	47.3	48.9	
Median	52.6	46.1	48.6	
IQR	23.2	23.2	25.5	
Range	31.7–70.0	13.0–72.8	13.0–72.8	
**Gender, n (%)**				0.192
Male	12 (75.0%)	12 (52.2%)	24 (61.5%)	
Female	4 (25.0%)	11 (47.8%)	15 (38.5%)	
**T Stage, n (%)**				**0.024**
T1	1 (6.2%)	2 (8.7%)	3 (7.7%)	
T2	5 (31.2%)	16 (69.6%)	21 (53.8%)	
T3	1 (6.2%)	2 (8.7%)	3 (7.7%)	
T4	9 (56.2%)	3 (13.0%)	12 (30.8%)	
**Size (cm)**				**0.013**
Mean	3.8	2.9	3.2	
Median	3.4	3.1	3.2	
IQR	1.9	1.2	1.3	
Range	1.5–6.2	1.4–4.2	1.4–6.2	
**Margins, n (%)**				**0.006**
Smooth	7 (43.8%)	20 (87.0%)	27 (69.2%)	
Irregular	0 (0.0%)	1 (4.3%)	1 (2.6%)	
Mixed	9 (56.2%)	2 (8.7%)	11 (28.2%)	
**Shape of tumor, n (%)**				0.212
Rounded/oblong	4 (25.0%)	9 (39.1%)	13 (33.3%)	
Molds to orbit	6 (37.5%)	11 (47.8%)	17 (43.6%)	
Infiltrative	6 (37.5%)	3 (13.0%)	9 (23.1%)	
**Tail, n (%)**				0.156
Absent	15 (93.8%)	16 (69.6%)	31 (79.5%)	
Grade 1	0 (0.0%)	2 (8.7%)	2 (5.1%)	
Grade 2	1 (6.2%)	1 (4.3%)	2 (5.1%)	
Thin dural enhancement	0 (0.0%)	4 (17.4%)	4 (10.3%)	
**Tumor through SOF, n (%)**				0.061
No	13 (81.2%)	23 (100.0%)	36 (92.3%)	
Yes	3 (18.8%)	0 (0.0%)	3 (7.7%)	
**Distance from posterior tumor to SOF (mm)**				**0.017**
Mean	7.6	15.2	12.1	
Median	7.5	15.0	10.0	
IQR	11.5	12.5	13.5	
Range	0.0–23.0	0.0–41.0	0.0–41.0	
**Mass effect on globe, n (%)**				1.000
No	1 (6.2%)	2 (8.7%)	3 (7.7%)	
Yes	15 (93.8%)	21 (91.3%)	36 (92.3%)	
**Indentation/flattening of globe, n (%)**				0.752
No	10 (62.5%)	13 (56.5%)	23 (59.0%)	
Yes	6 (37.5%)	10 (43.5%)	16 (41.0%)	
**Proptosis, n (%)**				0.370
No	1 (6.2%)	5 (21.7%)	6 (15.4%)	
Yes	15 (93.8%)	18 (78.3%)	33 (84.6%)	
**Bone remodeling, n (%)**				0.503
No	16 (100.0%)	21 (91.3%)	37 (94.9%)	
Yes	0 (0.0%)	2 (8.7%)	2 (5.1%)	
**Bone erosion, n (%)**				**0.027**
No	8 (50.0%)	20 (87.0%)	28 (71.8%)	
Yes	8 (50.0%)	3 (13.0%)	11 (28.2%)	
**Involvement of muscle cone, n (%)**				**0.049**
Extraconal only	4 (25.0%)	14 (60.9%)	18 (46.2%)	
Intraconal	12 (75.0%)	9 (39.1%)	21 (53.8%)	
**EOM involvement, n (%)**				**0.022**
Abut	12 (75.0%)	23 (100.0%)	35 (89.7%)	
Invade	4 (25.0%)	0 (0.0%)	4 (10.3%)	
**Optic nerve involvement, n (%)**				0.060
None	9 (56.2%)	20 (87.0%)	29 (74.4%)	
Abut	7 (43.8%)	3 (13.0%)	10 (25.6%)	
**Tumor crosses vertical midline, n (%)**				**0.008**
No	3 (18.8%)	15 (65.2%)	18 (46.2%)	
Yes	13 (81.2%)	8 (34.8%)	21 (53.8%)	
**Cavernous sinus involvement, n (%)**				0.162
No	14 (87.5%)	23 (100.0%)	37 (94.9%)	
Yes	2 (12.5%)	0 (0.0%)	2 (5.1%)	
**Temporal fossa involvement around zygoma, n (%)**				0.205
No	12 (75.0%)	21 (91.3%)	33 (84.6%)	
Yes	4 (25.0%)	2 (8.7%)	6 (15.4%)	
**Temporal fossa involvement through lateral wall, n (%)**				**0.013**
No	10 (62.5%)	22 (95.7%)	32 (82.1%)	
Yes	6 (37.5%)	1 (4.3%)	7 (17.9%)	
**Pterygopalatine fossa involvement, n (%)**				0.410
No	15 (93.8%)	23 (100.0%)	38 (97.4%)	
Yes	1 (6.2%)	0 (0.0%)	1 (2.6%)	
**Dura/brain involvement, n (%)**				0.064
None	11 (68.8%)	22 (95.7%)	33 (84.6%)	
Touch	3 (18.8%)	0 (0.0%)	3 (7.7%)	
Beyond	2 (12.5%)	1 (4.3%)	3 (7.7%)	
**Sinonasal involvement, n (%)**				0.061
No	13 (81.2%)	23 (100.0%)	36 (92.3%)	
Yes	3 (18.8%)	0 (0.0%)	3 (7.7%)	
**Perineural spread, n (%)**				0.410
No	15 (93.8%)	23 (100.0%)	38 (97.4%)	
Yes	1 (6.2%)	0 (0.0%)	1 (2.6%)	

Note: Bold *p*-values indicate statistical significance (*p* < 0.05).

## Data Availability

Data available upon request from corresponding author.
